# Therapeutic Drug Monitoring of the Newer Anti-Epilepsy Medications

**DOI:** 10.3390/ph3061908

**Published:** 2010-06-11

**Authors:** Matthew D. Krasowski

**Affiliations:** Department of Pathology, University of Iowa Hospitals and Clinics, 200 Hawkins Drive, RCP 6233, Iowa City, IA 52242, USA; E-Mail: mkrasows@healthcare.uiowa.edu; Tel.: +1-319-384-9380, Fax: +1-319-384-8051

**Keywords:** anticonvulsants, drug monitoring, drug toxicity, epilepsy, seizures

## Abstract

In the past twenty years, 14 new antiepileptic drugs have been approved for use in the United States and/or Europe. These drugs are eslicarbazepine acetate, felbamate, gabapentin, lacosamide, lamotrigine, levetiracetam, oxcarbazepine, pregabalin, rufinamide, stiripentol, tiagabine, topiramate, vigabatrin and zonisamide. In general, the clinical utility of therapeutic drug monitoring has not been established in clinical trials for these new anticonvulsants, and clear guidelines for drug monitoring have yet to be defined. The antiepileptic drugs with the strongest justifications for drug monitoring are lamotrigine, oxcarbazepine, stiripentol, and zonisamide. Stiripentol and tiagabine are strongly protein bound and are candidates for free drug monitoring. Therapeutic drug monitoring has lower utility for gabapentin, pregabalin, and vigabatrin. Measurement of salivary drug concentrations has potential utility for therapeutic drug monitoring of lamotrigine, levetiracetam, and topiramate. Therapeutic drug monitoring of the new antiepileptic drugs will be discussed in managing patients with epilepsy.

## 1. Background on Therapeutic Drug Monitoring of Antiepileptic Medications

Drugs used to prevent and treat seizures (antiepileptic drugs, AEDs) have been among the most common medications for which therapeutic drug monitoring (TDM) is performed [1,2]. Traditionally, TDM has been applied mainly to the 'older' or first-generation AEDs that have been on the market in the United States and Europe for several decades, namely carbamazepine, phenobarbital, phenytoin, primidone, and valproic acid. These first-generation AEDs in general have narrow therapeutic ranges and significant inter-individual variability in their pharmacokinetics (absorption, distribution, metabolism, and excretion). Somewhat surprisingly, given the common practice of utilizing TDM for AEDs, the evidence that TDM of AEDs significantly helps clinical management is mostly anecdotal and retrospective. Only two randomized, controlled studies of AED TDM have been conducted and neither showed clinical benefits. Both studies did show, however, that physicians often apply information from TDM incorrectly, diminishing the clinical impact of TDM [3,4]. Better education of medical practitioners on TDM is a priority for the future.

TDM of AEDs faces three main challenges [2]. Firstly, seizures occur irregularly, sometimes with long periods of time between episodes. Consequently, long-term observation of any therapy for seizures may be needed to assess clinical benefit. Secondly, some AEDs produce adverse effects that may be difficult to distinguish from the underlying neurologic disease. Lastly, there are no simple laboratory tests or diagnostic procedures that can assess the clinical efficacy of AEDs. Clinical observation and relatively labor-intensive procedures such as the electroencephalogram (EEG) remain the mainstays of clinical assessment.

In applying TDM to any drug, the most basic assumption is that the concentration being measured correlates with the concentration at the target site of action (e.g., ion channel in the brain). TDM is usually performed on serum or plasma, or occasionally on some other body fluid such as saliva, urine or cerebrospinal fluid. Factors that can negatively affect the correlation between clinical effect and serum/plasma concentration include tolerance to the drug, irreversibility of drug action and active metabolites. For drugs with active metabolites, TDM can include measurement of the concentrations of both parent drug and its metabolite(s) or just of the metabolite(s). As an example, TDM of oxcarbazepine focuses on 10-hydroxycarbazepine (major metabolite), as will be discussed below.

TDM of newer AEDs in saliva has not yet been widely applied [5], but has been studied for six drugs: gabapentin [6], lamotrigine [7], levetiracetam [8], oxcarbazepine (main metabolite 10-hydroxycarbazepine) [9], topiramate [10] and zonisamide [11]. Of these six drugs, gabapentin appears to be clearly unsuited for salivary concentration analysis due to the low concentration in saliva *versus* plasma (salivary concentrations are ~5–10% that of serum or plasma). General concerns with monitoring salivary concentrations are shorter half-life of some drugs in saliva compared to serum and difficulties in analyzing patients who have little saliva or viscous saliva. However, a key advantage of monitoring saliva is ease of collection, especially in the pediatric and geriatric populations. A study has demonstrated that salivary samples for monitoring AEDs can be collected by the patient and mailed to a clinical laboratory without significant degradation of sample [12].

## 2. Reasons for Applying TDM to AEDS

There are multiple reasons why TDM may be useful in the clinical management of AED therapy. A common reason is that the pharmacokinetics of the drug shows significant inter-individual variability [13,14,15]. If the pharmacokinetics is very consistent and predictable, then dosing of the drug can often be done without TDM. Metabolism (biotransformation) is a major pharmacokinetic factor that can affect AEDs. Variability in metabolism may be due to impaired organ function (typically kidney or liver), genetic factors (pharmacogenetics), or drug-drug or drug-food interactions. Several AEDs, namely carbamazepine, phenobarbital, and phenytoin, are well-known 'inducers' (stimulators) of 'drug-metabolizing' enzymes in the liver and other organs [16]. Carbamazepine, phenobarbital, and phenytoin act on nuclear hormone receptors such as the pregnane X receptor (PXR, NR1I2) or the constitutive androstane receptor (CAR, NR1I3). When activated by ligands such as carbamazepine, PXR and CAR increase the expression of cytochrome P450 (CYP) enzymes, phase II enzymes (e.g., glucuronidating enzymes), and efflux transporters, all of which can accelerate the elimination of AEDs and other drugs [17,18].

AEDs are often used in patients with some degree of renal impairment. Renal insufficiency can alter AED pharmacokinetics by decreased clearance of drug and/or metabolites, or by removal of drug during dialysis procedures. For some AEDs, there has been little investigation of the effect of dialysis on AED plasma concentration. In general, AEDs with low degrees of plasma protein binding are cleared more effectively by dialysis than those AEDs with high degrees of protein binding [19]. In the discussion of the specific AEDs in this review, studies of the effect of dialysis on AED are mentioned if available in the published literature.

Carbamazepine represents an example of a drug that shows 'autoinduction', namely that the metabolism of carbamazepine increases as the drug is used chronically [20]. This means that the carbamazepine dose needs to be increased over time to keep pace with the increases in metabolism, until the induction finally plateaus. Other known enzymes inducers include rifampin (a tuberculosis drug) and St. John's wort (a herbal antidepressant) [16]. Some drugs may also inhibit metabolism of AEDs (typically by blocking CYP enzyme catalytic activity), potentially leading to excessively high concentrations of drug unless the dose is reduced appropriately. As an example, valproic acid is an inhibitor of multiple liver enzymes and has been well-documented to cause drug-drug interactions with other AEDs [1]. Variability in pharmacokinetics may also occur due to alterations in drug absorption or distribution. AEDs that show variable and unpredictable pharmacokinetics are good candidates for TDM [2].

For some medications that are highly (>90%) bound to serum proteins, monitoring of free (unbound) drug concentrations may be clinically useful [21]. A number of factors may alter serum protein concentrations including liver disease, old age, and pregnancy. Concomitant medications (e.g., valproic acid) or endogenous substances may displace drugs from serum protein binding sites, potentially leading to higher free drug concentrations. Uremia, as may occur in renal failure, may also increase free AED concentrations by displacement from serum protein binding sites. Free drug concentrations are typically measured by analyzing the concentration of drug present in an ultrafiltrate of plasma or serum. The technical challenge is that free drug concentrations for drugs that are highly protein bound are substantially lower than total drug concentrations. Some analytical methods that may be suitable for measuring total drug concentrations may have insufficient analytical sensitivity to accurately measure the full range of clinically useful free drug concentrations [21]. In addition, the ultrafiltration process is not easily automated and thus adds manual processing time to the clinical laboratory analysis of AEDs that require determination of free drug concentration.

TDM may also be used to assess adherence (compliance) to therapy [2]. Given that AEDs may be prescribed for years, even in the absence of seizures, patients may skip doses or stop taking the medication altogether. The presence of adverse effects or cost of medications can be other reasons that patients stop taking their medication.

## 3. The Newer Generation of AEDs

In the last twenty years, 14 new AEDs have entered the market in the United States and/or Europe [22,23]. These drugs are eslicarbazepine acetate, felbamate, gabapentin, lacosamide, lamotrigine, levetiracetam, oxcarbazepine, pregabalin, rufinamide, stiripentol, tiagabine, topiramate, vigabatrin and zonisamide. Eslicarbazepine acetate, lacosamide, rufinamide, and stiripentol are not yet approved in the United States. The newer AEDs are sometimes characterized as second- or third-generation drugs. In comparison to the older AEDs, the newer agents often have wider therapeutic ranges and fewer serious adverse effects. Like some of the older AEDs, the newer agents may also be used for other conditions such as bipolar disorder ('manic depression'), chronic pain syndromes (e.g., fibromyalgia, trigeminal neuralgia), or migraine headaches [22,24]. This manuscript focuses on TDM of the newer AEDs in treatment of epilepsy, emphasizing whether the pharmacokinetics and clinical effects of the drug make TDM useful.

## 4. The Challenge of Establishing Reference Ranges for AEDs

Reference ranges for the newer AEDs have generally been difficult to establish [2]. These drugs are usually effective over a wide range of serum/plasma concentrations but with substantial inter-individual variation in response. Ideally, TDM would guide physicians towards serum/plasma concentrations that optimize seizure control, while avoiding or at least minimizing toxic effects. The 'reference range' of an AED can be defined by two limits—a lower limit below which therapeutic effect is unlikely and an upper limit above which toxicity is likely [2]. However, any particular individual may have good clinical response at AED concentrations outside the reference range. In addition, reference ranges may vary with different types of seizures, or when AEDs are used to treat other clinical conditions such as neuropathic pain or bipolar disorder. For example, the original reference range for an AED may have been derived from studies of patients with refractory epilepsy, a population that may respond quite differently from patients with more easily treatable disease. Furthermore, many of the newer AEDs were first studied as adjunctive therapy and not as monotherapy. Perucca has promoted the concept of 'individual therapeutic concentrations' for AEDs [25] wherein a patient is treated until good seizure control is achieved. The serum/plasma concentration is determined and then serves as the patient's individual therapeutic concentration. TDM can then be applied periodically to determine whether the concentration is staying near the individual therapeutic concentration [25,26]. Drug monitoring can be especially important when changes in the patient occur that alter AED pharmacokinetics, e.g., pregnancy, impaired kidney or liver function, or concomitant therapy with enzyme-inducing or -inhibiting drugs. One limitation of the individual therapeutic concentration is that changes in the underlying seizure condition may require establishment of a new individual therapeutic concentration.

**Figure 1:**
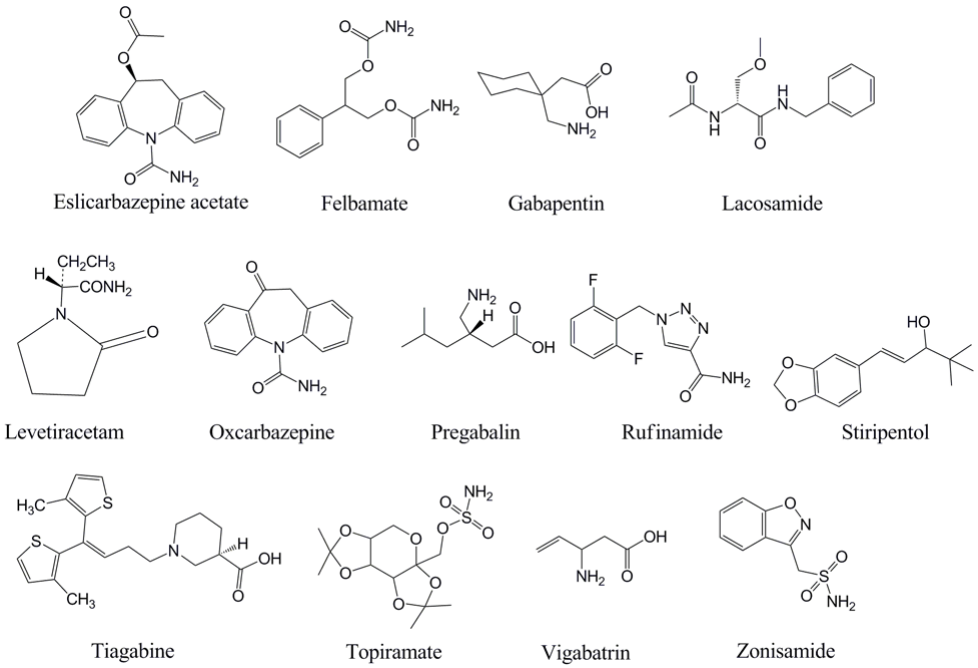
Chemical structures of the antiepileptic drugs discussed in this review.

With the background and theory on TDM above, each of the newer AEDs will be discussed in turn with regard to TDM. This manuscript focuses on monitoring of serum or plasma, although as mentioned above there has been some clinical application of measuring AED concentrations in other fluids (e.g., saliva) [5]. For discussion of the analytical methods that can be used for measurement of AED serum/plasma concentrations, selected numbers of representative references are cited. Table 1 summarizes the pharmacokinetic properties of the newer AEDs, while Table 2 presents a summary of the factors that influence the use and interpretation of TDM for the AEDs. The chemical structures of the AEDs are in Figure 1.

**Table 1:** Pharmacokinetic properties and reference ranges for the AEDs.

Drug	Oral Bioavailability (%)	Serum protein binding (%)	Time to peak concentration (h)	Half-life in Absence of Concomitant Enzyme Inducers^a^	Half-life in Presence of Concomitant Enzyme Inducers^a^	Reference Range in Serum (mg/L)^f^
Eslicarbazepine acetate	≥80	30	1–4	20–24	20–24	Not established
Felbamate	>90	25	2–6	16–22	10–18	30–60
Gabapentin	<60	0	2–3	5–9	5–9	2–20
Lacosamide	≥95	15	0.5–4	12–13	12–13	5–10
Lamotrigine	≥95	55	1–3	15–35^b^	8–20	3–14
Levetiracetam	≥95	0	1	6–8	6–8	12–46
Oxcarbazepine^c^	90	40	3–6	8–15	7–12	3–35
Pregabalin	≥90	0	1–2	5–7	5–7	2.8–8.3
Rufinamide	85	30	5–6	8–12	≤8	Not established
Stiripentol^d^	≥90	99	1–2	Variable^e^	Variable^e^	4–22
Tiagabine^d^	≥90	96	1–2	5–9	2–4	0.02–0.2
Topiramate	≥80	15	2–4	20–30	10–15	5–20
Vigabatrin	≥60	0	1–2	5–8	5–8	0.8–36
Zonisamide	≥65	50	2–5	50–70	25–35	10–40

^a^ Enzyme inducers include carbamazepine, phenobarbital, phenytoin, rifampicin, and St. John's wort. References for drugs whose half-lives are altered in patients receiving liver enzyme inducers: felbamate [36], lamotrigine [37], oxcarbazepine [38], rufinamide [39], tiagabine [40], topiramate [41] and zonisamide [15]. ^b^ Half-life increases to 30–90 h during concomitant therapy with valproic acid (enzyme inhibitor). ^c^ All parameters refer to the active metabolite 10-hydroxycarbazepine. ^d^ Monitoring of free drug may be useful for these drugs. ^e^ Drug shows zero-order elimination kinetics. ^f^ References for reference ranges: felbamate [42,43], gabapentin [44], lacosamide [45], lamotrigine [46], levetiracetam [47], oxcarbazepine (10-hydroxycarbazepine metabolite) [48], pregabalin [2], stiripentol [49], tiagabine [50], topiramate [51], vigabatrin [23], zonisamide [52].

**Table 2:** Summary of factors that influence use and interpretation of TDM for newer AEDs.

Drug	Factors that Favor Use of TDM	Factors that May Limit Use of TDM and/or Complicate Interpretation
Eslicarbazepine acetate	Auto-induction with chronic dosing	Has active metabolite (oxcarbazepine)
	Liver failure	
Felbamate	Variable metabolism	Unclear toxic concentrations
	Potential for severe toxicity	
Gabapentin	Variable absorption	Wide range of clinically effective serum concentrations
	Renal failure	
		Low incidence of toxicity
Lacosamide	Liver failure	Generally predictable pharmacokinetics Drug-drug interactions uncommon
	Renal failure	
Lamotrigine	Variable metabolism	
	Common drug-drug interactions	
	Well-defined toxic concentrations	
	Common use in pregnancy	
Levetiracetam	Renal failure	Wide range of clinically effective serum concentrations
		Low incidence of toxicity
Oxcarbazepine	Variable metabolism	
	Well-defined toxic concentrations	
Pregabalin	Variable absorption	Wide range of clinically effective serum concentrations
	Renal failure	
		Low incidence of toxicityShort half-life
Rufinamide	Variable absorption	
	Common drug-drug interactions Renal failure	
Stiripentol	Extensive first-pass metabolism High serum protein binding	High serum protein binding can complicate interpretation of total drug concentrations (free drug levels may be helpful)
	Zero-order elimination kinetics	
Tiagabine	High serum protein bindingLiver failure	High serum protein binding can complicate interpretation of total drug concentrations (free drug levels may be helpful)
	Common drug-drug interactions	
Topiramate	Common drug-drug interactions	
Vigabatrin	Renal failure	Poor correlation of serum concentrations and therapeutic effect (irreversible effect)
Zonisamide	Variable metabolism	
	Common drug-drug interactions	
	Well-defined toxic concentrations	

## 5. Eslicarbazepine Acetate

Eslicarbazepine acetate [(*S*)-licarbazepine acetate] is a pro-drug that is rapidly metabolized by liver esterases to form eslicarbazepine, a compound that inhibits voltage-gated sodium channels [27]. Following oral administration, approximately 95% of eslicarbazepine acetate is converted to eslicarbazepine in plasma [28]. TDM focuses on eslicarbazepine (active metabolite). Minor metabolites of eslicarbazepine acetate are oxcarbazepine (also used as an AED) and (*R*)-licarbazepine. Unlike carbamazepine, eslicarbazepine acetate is not converted to carbamazepine-10,11-epoxide and does not exhibit auto-induction in metabolism. Eslicarbazepine has low (~30%) binding to serum proteins and an elimination half-life of 20–24 h during chronic administration [29]. Eslicarbazepine has low potential for drug-drug interactions [30,31]. The pharmacokinetics of eslicarbazepine are not significantly affected by mild to moderate hepatic failure [32]. Clearance of eslicarbazepine and minor metabolites of eslicarbazepine acetate is predominantly by the kidney, with significant increases in area under the curve (AUC) in patients with moderate or severe renal failure. Hemodialysis effectively clears eslicarbazepine and other metabolites of eslicarbazepine acetate [33].

Overall, TDM has a minimal role in the therapeutic use of eslicarbazepine due to the relatively predictable pharmacokinetics of the drug. An enantioselective high-performance liquid chromatography-ultraviolet (HPLC-UV) method has been developed for the specific monitoring of eslicarbazepine and its metabolites [34]. It remains to be seen if eslicarbazepine acetate, like carbamazepine, has a therapeutic role in the management of bipolar disorder [35].

## 6. Felbamate

Felbamate was approved in 1993 in the United States for the treatment of partial seizures in adults and for Lennox-Gastaut Syndrome, a type of childhood epilepsy that is often refractory to AED therapy [53]. By 1994, cases of aplastic anemia and later severe liver failure were identified and associated with felbamate therapy. The drug has remained on the market but with revised labeling and much restricted use [53]. In terms of pharmacokinetics, felbamate has high bioavailability (>90%). Approximately 50% of the parent drug is ultimately metabolized by the liver to inactive metabolites [54]. It is suspected that one or more of the metabolites mediates the rare but serious adverse effects [55]. Inducers of liver metabolism (e.g., carbamazepine, phenytoin, phenobarbital, rifampin, St. John's wort) increase the metabolism of felbamate [56], while valproic acid inhibits the metabolism [57]. Felbamate does not have a clear reference range but typical doses used in epilepsy management usually result in serum/plasma concentrations of 30–60 mg/L [43]. The clearance of felbamate is 20–65% higher in children than in adults [14].

The variable metabolism of felbamate and differences between children and adults in clearance suggest that TDM may be helpful in felbamate therapy. Multiple analytical methodologies have been reported for the measurement of felbamate in plasma/serum including HPLC [58,59], gas chromatography (GC) [60] and capillary electrophoresis [61]. However, the rare adverse effects have severely limited the use of felbamate [53]. Close monitoring of blood counts and liver function are advised during felbamate therapy.

## 7. Gabapentin

Gabapentin was originally approved in 1994 in the United States for the treatment in epilepsy but has achieved greater popularity as an adjunctive therapy for chronic pain. Although structurally related to the neurotransmitter γ-aminobutyric acid (GABA), gabapentin does not appear to interact with GABA receptors in the brain or spinal cord [22,62]. Gabapentin is rapidly absorbed by the *L*-amino acid transport system [63]. A study published in 1998 showed a decrease in bioavailability at doses of 4800 mg/day of gabapentin as compared to lower doses, suggesting possible saturability of the *L*-amino acid transport system [64]. However, a later study demonstrated linear absorption up to 4800 mg/day [6]. Salivary concentrations of gabapentin are only 5–10% those in plasma, limiting the utility of salivary gabapentin concentrations for TDM [6]. Gabapentin is not metabolized and shows little binding to serum proteins [63]. The majority of drug is excreted renally and the half-life of the drug increases in renal failure [62]. Gabapentin is effectively cleared by hemodialysis [65]. A wide range of serum/plasma concentrations are associated with clinical effect [66] although effective control of seizures typically requires concentrations above 2 mg/L [67]. An approximate reference range of 2–20 mg/L has been proposed [44]. Multiple analytical methodologies have been reported for the measurement of gabapentin in plasma/serum including HPLC [68,69], HPLC-tandem mass spectrometry (LC/MS/MS) [70], gas chromatography/mass spectrometry (GC/MS) [71] and GC/MS/MS [72].

## 8. Lacosamide

Lacosamide is a novel functionalized amino acid whose mechanism of action is thought to involve enhancement of slow inactivation of sodium channels [73]. Lacosamide was approved in Europe in September 2008 for partial-onset seizures in patients 16 years and older [74]. Lacosamide has a bioavailability of ~100% with minimal first-pass metabolism and serum protein binding [75]. Approximately 40% of the drug is ultimately excreted by the kidney with the remainder cleared by metabolism. Although the effect of hemodialysis on lacosamide pharmacokinetics has not been reported, the low plasma protein binding of lacosamide suggests that this drug should be effectively cleared by dialysis [19]. CYP2C19 (an enzyme that can show pharmacogenetic variation) catalyzes the metabolism of lacosamide to an inactive metabolite. The half-life of lacosamide is approximately 12 hours. Clinically significant drug-drug interactions involving lacosamide are minimal [76], including a detailed study showing a lack of interaction between carbamazepine and lacosamide [77]. Both HPLC [78] and LC/MS/MS [79] procedures for measuring lacosamide concentrations in plasma or serum have been reported. Overall, lacosamide has predictable pharmacokinetics with no clinically significant differences in pharmacokinetics between children, young adults, and elderly patients. Other than to establish individualized references ranges, TDM of lacosamide is likely best focused on patients with liver and/or kidney failure [80].

## 9. Lamotrigine

Lamotrigine was approved by the United States Food and Drug Administration (FDA) in late 1994 as an adjunctive therapy for partial seizures [22]. Lamotrigine has since gained indications as monotherapy for partial seizures and also as a treatment for bipolar disorder [1,2]. Lamotrigine has also accumulated a solid safety record in pregnancy, leading to the common use of this AED in pregnant women with epilepsy [81,82]. Lamotrigine is rapidly and completely absorbed from the gastrointestinal tract and is only 50–60% bound to serum proteins. Lamotrigine distributes into saliva, with salivary lamotrigine concentrations being on average approximately 0.4–0.5 that of serum concentrations in patients receiving chronic lamotrigine therapy. Salivary lamotrigine concentrations correlate well with those in serum, which makes saliva an alternative sample to perform TDM [83,84]. The parent drug is extensively metabolized, mainly by glucuronidation to an inactive metabolite [85]. Similar to carbamazepine, the metabolism of lamotrigine shows the phenomenon of autoinduction. For most patients, autoinduction is complete within two weeks, with a ~20% reduction in steady-state serum/plasma concentrations if the dose is not changed [86]. The metabolism of lamotrigine is significantly affected by concomitant use of classic liver enzyme inducers [85]. Oral contraceptives containing ethinyl estradiol also significantly reduce the serum concentrations of lamotrigine [87,88]. The serum half-life of lamotrigrine typically is 15–35 h when used as monotherapy but only 8–20 h when use concomitantly with a liver enzyme inducers, and up to 60 h when used together with valproic acid, a CYP enzyme inhibitor [85]. The half-life of lamotrigine increases to ~50 h in patients with severe renal failure. Lamotrigine is effectively cleared by hemodialysis [89]. The clearance of lamotrigine is higher in children [14,46] and markedly higher (~300%) in pregnancy [14]. There is not a tight relationship between clinical response and serum/plasma concentrations [46], but a reference range of 3–14 mg/L has been proposed for refractory epilepsy therapy [90]. The incidence of toxic effects are significantly increased when serum/plasma concentrations exceed 15 mg/L [90].

Three factors of lamotrigine make TDM clinically useful. First, the drug shows significant interindividual variation in dose *versus* serum/plasma, in large part due to multiple factors that can affect liver metabolism of the drug. Second, the clearance of lamotrigine varies substantially during pregnancy and also across age groups [91]. Lastly, there is a fairly clear concentration threshold above which toxic side effects become more common [90,91]. Multiple analytical methodologies have been reported for the measurement of lamotrigine in plasma/serum including HPLC [92,93,94], radioimmunoassay [95], homogeneous immunoassay [96], immunofluorometric assay [97], capillary electrophoresis [98], capillary zone electrophoresis-electrospray ionization-mass spectrometry [99], gas chromatography (GC) with a nitrogen-phosphorus detector [100], GC/MS [101], LC/MS [102], LC/MS/MS [103] and micellar electrokinetic capillary chromatography [104].

## 10. Levetiracetam

Levetiracetam is a novel anticonvulsant [105] whose mechanism of action is thought to involve binding of the synaptic vesicle protein SV2A, a protein involved in neurotransmitter vesicle exocytosis [106]. Levetiracetam is rapidly and nearly completely absorbed following oral administration, although the rate of oral absorption is slowed by co-ingestion with food [107]. Levetiracetam distributes well into saliva, with salivary levetiracetam concentrations being on average slightly higher than serum concentrations in patients receiving chronic levetiracetam therapy [108]. Salivary levetiracetam concentrations correlate well with those in serum, which makes saliva an alternative sample to perform TDM [109]. Levetiracetam does not bind serum proteins and has linear pharmacokinetics. Approximately 100% of the absorbed drug is excreted by the kidneys [110], with approximately two-thirds as the parent drug and the remainder as the metabolite LO57, formed by hydrolysis in the blood [111]. Although the effect of hemodialysis on levetiracetam pharmacokinetics has not been reported, the low plasma protein binding of levetiracetam suggests that this drug should be effectively cleared by dialysis [19]. In performing TDM, it is important to separate serum or plasma from whole blood rapidly, as artefactual hydrolysis of levetiracetam can occur in the blood tube [111]. Because levetiracetam is not metabolized by the liver, significant drug-drug interactions are uncommon [76]. The serum half-life of levetiracetam is longer (16–18 h) in neonates compared to adults (6–8 h) [2]. An approximately 60% decrease in serum concentrations is observed in pregnancy [82]. From evaluation of 470 patients in a specialty epilepsy clinic, a reference range of 12–46 mg/L has been proposed [47]. Multiple analytical methodologies have been reported for the measurement of levetiracetam in plasma/serum including HPLC [112,113], GC [114,115], GC/MS [116], LC/MS/MS [117], microemulsion electrokinetic chromatography [118] and capillary electrophoresis [119]. Other than to evaluate potential toxicity or to assess compliance, the value of TDM for levetiracetam is mostly in adjusting dosage for renal insufficiency [120].

## 11. Oxcarbazepine

Oxcarbazepine is structurally related to carbamazepine but does not produce nearly as much induction of liver enzymes as carbamazepine and also shows a lower incidence of agranulocytosis [38,121]. Oxcarbazepine is rapidly and completely absorbed [38] and metabolized via 10-keto reduction to its monohydroxy derivative 10-hydroxycarbazepine. 10-Hydroxycarbazepine has equal potency to oxcarbazepine in antiseizure activity, but accumulates to higher concentrations in serum [122]. 10-Hydroxycarbazepine also distributes into saliva, with salivary 10-hydroxycarbazepine concentrations ranging from 0.3 to 1.7 that of serum concentrations [9]. However, the use of saliva as an alternative specimen type for TDM of oxcarbazepine is limited by dose-dependent variations in the correlation between 10-hydroxycarbazepine saliva and serum concentrations and a shorter half-life of 10-hydroxycarbazepine in saliva as compared to serum [9,123].

For the purposes of TDM, oxcarbazepine is treated like a pro-drug, with monitoring focusing on the monohydroxy metabolite as the main mediator of the antiseizure effects [2]. 10-Hydroxycarbazepine is further metabolized, primarily by glucuronidation. The clearance of 10-hydroxycarbazepine is reduced in the elderly [14] and also in the setting of renal insufficiency [124]. There is little data on the effect of hemodialysis on oxcarbazepine or 10-hydroxycarbazepine except for a case report of the successful treatment of massive oxcarbazepine overdose by hemodialysis [125]. The clearance of 10-hydroxycarbazepine is increased in pregnancy [126] and in patients taking liver enzyme-inducing drugs [38]. Young children require higher doses of oxcarbazepine per body weight than adults [127].

In a study of 947 patients, a wide range of 10-hydroxycarbazepine serum concentrations (3–35 mg/L) were observed to be clinically effective in seizure treatment [48], with toxic side effects being more common at serum/plasma concentrations of 35 mg/L or higher [128]. Multiple analytical methodologies have been reported for the measurement of 10-hydroxycarbazepine in plasma/serum including GC [129], GC/MS [130], HPLC [131,132], LC/MS [133], LC/MS/MS [134] and micellar electrokinetic chromatography [135]. TDM is justified when changes are expected that might alter 10-hydroxycarbazepine clearance including changes in renal function, pregnancy, and concomitant use of liver enzyme-inducing drugs.

## 12. Pregabalin

Pregabalin was designed to be a more potent analog of gabapentin [136]. Like gabapentin, pregabalin has shown effectiveness in treating chronic pain and additionally gained an indication in the United States for the treatment of fibromyalgia [22]. Pregabalin has predictable pharmacokinetics with excellent bioavailability [137], essentially no metabolism, no reported drug-drug interactions and minimal binding to serum proteins. The majority of the absorbed dose (~98%) is excreted unchanged in the urine with a clearance that approximates glomerular filtration rate (GFR) [138]. Renal failure patients require reduced dosage and it is also seems prudent to consider age-related changes in renal function in pharmacotherapy with gabapentin [139]. Pregabalin is effectively cleared by hemodialysis [139]. No clear reference range has been established but an approximate range of 2.8–8.3 mg/L has been proposed [2]. The short half-life of pregabalin (4.6–6.8 h) [140] means that care must be taken in drawing blood for TDM. Similar to gabapentin, other than to adjust dosing during renal failure or to assess compliance, pregabalin is not a good candidate for routine TDM. Plasma and serum concentrations of pregabalin can be determined by HPLC following derivitization [141] or by LC/MS/MS [142].

## 13. Rufinamide

Rufinamide is a novel anticonvulsant whose mechanism of action is to prolong the inactivated state of voltage-gated sodium channels [143]. Rufinamide was approved for use in Europe in January 2007 and by the FDA in the United States in December 2008 for Lennox-Gastaut syndrome. Rufinamide is well-absorbed (~85%) following oral administration, with the absorption enhanced significantly when the drug is taken together with food [39]. The peak exposure (C_max_) to rufinamide may increase up to 50% when taken with food as compared to an empty stomach. For this reason, it is advised that patients consistently take rufinamide in the same temporal relation to meals. Rufinamide is extensively metabolized with only trace amounts of the parent drug being recovered in urine and feces. The primary route of metabolism is not via CYP enzymes but instead from carboxyesterase-mediated enzymatic hydrolysis to form an inactive derivative that is excreted in the urine.

The metabolism of rufinamide is accelerated by classic liver enzyme inducers such as carbamazepine, and rifampin [39]. The clearance of rufinamide is not affected much by impaired renal function; however, increased doses of rufinamide are generally needed in patients receiving hemodialysis due to removal of the drug by the dialysis procedure. TDM for rufinamide can be useful because serum levels correlate well with seizure control, allowing for determination of an individual therapeutic concentration [39,143,144]. However, there is currently insufficient data to define a general therapeutic range for rufinamide [39]. Rufinamide serum or plasma concentrations can be determined by HPLC [145]. Monitoring of serum levels can be especially helpful in patients taking concomitant liver enzyme inducers or who are receiving hemodialysis.

## 14. Stiripentol

Stiripentol is an AED that inhibits GABA reuptake and also produces barbiturate-like positive allosteric modulation of GABA_A_ receptors [146,147]. Stiripentol was originally approved in Europe in 2001. Stiripentol is rapidly absorbed following oral administration but has high first-pass metabolism and thus low bioavailability. There are at least 13 metabolites of stiripentol in humans involving 5 or more different metabolic pathways. A major challenge to the dosing of stiripentol is that the drug shows non-linear pharmacokinetics, with a marked decrease in clearance with increased dosage [148]. Stiripentol is also highly (>99%) protein bound which likely limits clearance of this drug by hemodialysis procedures [19]. The reference range for stiripentol is not well-defined but serum concentrations of 4–22 mg/L correlate with control of absence seizures in children [49].

The complex pharmacokinetics of stiripentol (non-linear pharmacokinetics, high serum protein binding, extensive metabolism) resemble those of the classic AED phenytoin [144]. Monitoring of the free drug fraction of stiripentol would be theoretically advantageous although methods to measure free fractions have not yet been reported. Care must also be taken with the use of stiripentol with carbamazepine, clobazam, phenobarbital, phenytoin, and valproic because stiripentol inhibits the metabolism of these drugs and/or their metabolites [149,150]. An HPLC method has been reported for the analysis of stiripentol concentrations in plasma or serum [151].

## 15. Tiagabine

Tiagabine is currently infrequently used in the United States and Europe [22]. The major limitation of tiagabine is a propensity to cause non-convulsive status epilepticus which has been well-documented in many case reports and retrospective studies [152,153,154]. The mechanism of anti-convulsive action of tiagabine is not clear although inhibition of GABA reuptake has been proposed [22]. Tiagabine is rapidly absorbed with excellent bioavailability [155]. Unlike many of the other newer anticonvulsants, tiagabine is highly bound to proteins (>96%). Valproic acid can displace tiagabine from plasma protein binding sites, leading to increased free concentrations of tiagabine [156]. Tiagabine is extensively metabolized with less than 1% of the absorbed parent drug excreted unchanged [50,155]. The metabolism of tiagabine is increased during concomitant therapy with classic liver enzyme inducers. Renal impairment has no significant effect on the pharmacokinetics of tiagabine [157]. The serum half-life is 2–4 h in patients receiving enzyme inducers and 5–9 h in those not receiving enzyme inducers [40]. The serum half-life increases to 12–16 h in severe liver failure [158]. Children have higher clearance than adults [159].

The inter-individual variation in liver metabolism makes tiagabine a strong candidate for TDM. However, the relatively short half-life of tiagabine under most conditions means that care must be taken in drawing blood for TDM, with trough levels obtained if feasible. The high binding to serum proteins further suggests that measurement of free drug concentrations may be useful [21]. However, there has been little investigation of the relationship between serum/plasma concentrations and therapeutic efficacy [2]. A broad reference range of 20–200 ng/mL has been proposed [50]. Analytical issues have been a challenging problem in measurement of tiagabine serum/plasma concentrations, with some assays not achieving a low enough limit of sensitivity to measure the full range of clinically relevant free drug concentrations [160]. Multiple analytical methodologies have been reported for the measurement of tiagabine in plasma/serum including GC/MS [161] and HPLC [162]. Research aimed at development of improved methodology for measurement of free drug concentrations may be stimulated if the clinical popularity of tiagabine increases in the future.

## 16. Topiramate

Topiramate has approval for treatment of epilepsy of children and adults, and also for the treatment of migraine headaches [22]. Following oral administration, topiramate is absorbed rapidly with a high bioavailability (~80%) and low binding to serum proteins [163]. Topiramate distributes into saliva, with salivary topiramate concentrations being on average approximately 0.9 that of serum concentrations in patients receiving chronic topiramate therapy. Salivary topiramate concentrations correlate well with those in serum, which makes saliva an alternative sample to perform TDM [10]. Approximately 50% of the absorbed dose is metabolized by the liver, with an increase in metabolism seen in patients concomitantly receiving therapy with classic enzyme inducers. Enzyme inducers can decrease the serum half-life from 20–30 h to approximately 12 h [41,164]. Children generally eliminate topiramate faster than adults [14,165]. Although the effect of hemodialysis on topiramate pharmacokinetics has not been reported, the low plasma protein binding of topiramate suggests that this drug should be effectively cleared by dialysis [19]. A reference range of 5–20 mg/L has been proposed for topiramate for epilepsy therapy [51]. The value of TDM of topiramate is mainly due to inter-individual variation in metabolism. Multiple analytical methodologies have been reported for the measurement of topiramate in plasma/serum including GC [166], GC/MS [167], HPLC [168], LC/MS [169,170], LC/MS/MS [171] and immunoassay [172,173].

## 17. Vigabatrin

Vigabatrin is an irreversible inhibitor of GABA transaminase, an enzyme that catalyzes the elimination of GABA [174,175]. The drug is supplied as a racemic mixture, with the *S*(+) enantiomer being active and *R*(-) enantiomer being therapeutically inactive [175]. The drug has high bioavailability (60–80%) [176], low binding to serum proteins and is primarily excreted unchanged in the urine [174]. Clearance of vigabatrin increased during hemodialysis [177]. Doses of vigabatrin generally need to be decreased during renal failure [174].

The irreversible effect of vigabatrin on its molecular target undermines one of the principle assumptions of TDM, namely that the concentration in serum/plasma clearly correlates with that at the target site of action. This may be one reason why a wide range of trough serum/plasma concentrations (0.8–36 mg/L) have been found in patients successfully treated with vigabatrin [23]. Multiple analytical methodologies have been reported for the measurement of vigabatrin in plasma/serum including capillary electrophoresis [178], GC/MS [71] and HPLC [179]. Other than to assess compliance or possible drug overdose, there is little justification for monitoring of vigabatrin plasma/serum concentrations [23].

## 18. Zonisamide

Zonisamide is approved in the United States for adjunctive treatment of partial seizures but is also used 'off-label' for bipolar disorder, chronic pain, and migraine headaches [22,52]. Zonisamide is also marketed in Asia and Europe. Zonisamide is rapidly absorbed after oral administration and is only 40–60% bound to serum proteins. Zonisamide distributes into saliva, but there is only limited data published on the relation between salivary and serum zonisamide concentrations [11]. Zonisamide displays linear pharmacokinetics but is extensively metabolized by oxidation, acetylation, and other pathways [180]. CYP3A4, the major liver drug-metabolizing enzyme in most people, is responsible for some of the metabolism of zonisamide. The metabolism of zonisamide can be significantly affected by CYP enzyme inhibitors (e.g., cimetidine, erythromycin, ketoconazole, valproic acid) and inducers. The serum half-life of zonisamide is approximately 50–70 h for patients receiving zonisamide as monotherapy but decreases to 25–35 h in patients concomitantly taking enzyme inducers. Conversely, liver enzyme inhibitors such as valproic acid and ketoconazole may prolong zonisamide half-life [15]. Zonisamide is effectively cleared by hemodialysis [181]. Children require higher doses by weight than adults [14]. Toxic side effects are uncommon at serum concentrations below 30 mg/L [182]. A serum/plasma reference range of 10–40 mg/L has been proposed for seizure management [52,183]. Multiple analytical methodologies have been reported for the measurement of zonisamide in plasma/serum including HPLC [184,185], LC/MS [186] and micellar electrokinetic capillary electrophoresis [187]. The inter-individual variability in metabolism of zonisamide, especially seen in those receiving concomitant therapy with other drugs that can affect liver enzyme expression, makes zonisamide an attractive candidate for TDM.

## 19. Summary and Further Applications

The newer generation of AEDs offers attractive pharmacological alternatives to the traditional AEDs for treatment of epilepsy and other disorders such as chronic pain, migraine headaches, and fibromyalgia. The newer AEDs generally have fewer adverse effects and wider therapeutic margins. The strongest cases for routine TDM can be made for lamotrigine, oxcarbazepine (mono-hydroxy metabolite), stiripentol, tiagabine, and zonisamide, mainly due to inter-individual variation in metabolism and clearance. For other drugs, TDM may be clinically useful to assess adherence or to adjust dosing in organ failure. Generalized reference ranges have yet to be proposed for some newer AEDs; however, even in the absence of generalized reference ranges, individual reference ranges can be established for patients treated with newer AEDs, allowing for expanded use of TDM. Future research is needed to better define reference ranges and to better document the value of TDM in clinical practice.
